# Presence of Myeloperoxidase in Lamellar Tissue of Horses Induced by an Euglycemic Hyperinsulinemic Clamp

**DOI:** 10.3389/fvets.2022.846835

**Published:** 2022-03-11

**Authors:** Nazare Storms, Carlos Medina Torres, Thierry Franck, Albert Sole Guitart, Geoffroy de la Rebière, Didier Serteyn

**Affiliations:** ^1^Department of Equine Surgery, University of Liège, Liège, Belgium; ^2^School of Veterinary Science, The University of Queensland, Gatton, QLD, Australia; ^3^Center for Oxygen Research and Development, FARAH, University of Liège, Liège, Belgium

**Keywords:** horse, myeloperoxidase, laminitis, insulin, neutrophils, metabolic disease

## Abstract

Laminitis is a pathology of the equine digit leading to a failure of the dermo-epidermal interface. Neutrophil activation is recognized as a major factor in SIRS-associated laminitis. Less is known about the role of neutrophil activation in laminitis associated with metabolic disorders. The aim of this descriptive study was to observe whether myeloperoxidase is increased in the laminae during early stage laminitis in three horses subjected to a prolonged euglycemic hyperinsulinemic clamp (pEHC). After 48 h of pEHC-treatment, horses were subjected to euthanasia. Two healthy horses are used as control. Histological sections of lamellar tissue from all horses were immunohistochemically stained for myeloperoxidase and counterstained with hematoxylin-eosin. Histopathological changes that characterize insulin-induced laminitis and increased presence of myeloperoxidase, especially in the dermal lamellae, were increased in histologic sections of pEHC-treated horses. Neutrophil myeloperoxidase release may contribute to the pathophysiology of endocrinopathic laminitis.

## Introduction

Equine laminitis is a pathology of the digit resulting in severe lameness and ultimately displacement of the distal phalanx. This occurs due to structural failure with loss of integrity of the lamellae attaching the hoof wall to the distal phalanx (1). Inflammation seems to play a central role in the pathogenesis of laminitis (2). In cases of sepsis, local infection is accompanied by systemic neutrophil activation. Systemic neutrophil activation is also encountered in equine laminitis, as demonstrated by the up-regulation of cytokine expression, the dynamic changes in blood neutrophil phenotype, the formation of neutrophil-platelet aggregates and the infiltration of inflammatory cells (3–6). The systemic and the local inflammatory responses have largely been described in previously developed laminitis models such as Carbohydrate Overload (CHO), Oligofructose (OF) and Black Walnut Heartwood Extract (BWHE). This underscores the major role of neutrophil activation (7–10). Using myeloperoxidase (MPO) as a marker of neutrophil activity, Riggs and colleagues confirmed neutrophil activation in blood, skin, and lamellar tissue starting from one and measured to 12 h following BWE administration (9).

MPO is a hemic enzyme, responsible for the direct or indirect synthesis of many oxidizing species that participate in host defense mechanisms. MPO has dual peroxidase and chlorination activity, and their derived products (e.g., HOCl, nitrogen dioxide) are able to induce chlorination, nitration, and oxidation of protein residues. Hypochlorous acid (HOCl) produced *via* the chlorination activity of MPO, using hydrogen peroxide (H_2_O_2_) and a chloride anion is recognized as a powerful oxidizing agent necessary for the destruction of micro-organisms in the phagolysosome. When the inflammatory reaction becomes uncontrolled, excessive neutrophil degranulation or death induces significant MPO release into the extracellular environment, and the oxidant products derived from its activity can induce cell and tissue damage (11–13).

Laminitis has also been associated with equine metabolic disorders, such as obesity, pituitary pars intermedia dysfunction, and equine metabolic syndrome (EMS). The primary characteristic of these pathologies is the development of insulin resistance, characterized by hyperinsulinemia with eu- or hyperglycemia and a subsequent chronic pro-inflammatory state (14). As in the human metabolic syndrome where increased adipose tissue mass amplifies the secretion of proinflammatory adipokines that decrease insulin sensitivity, induce oxidative stress, and impair microvascular function; the EMS is associated with similar risk factors and characterized by regional adiposity, hyperinsulinemia, insulin resistance, hypertriglyceridemia, and recurrent laminitis (15, 16).

An experimental model of laminitis showed that healthy Standardbred horses subjected to prolonged hyperinsulinaemia develop laminitis within 48 h (17). Furthermore, natural cases of equine endocrinopathic laminitis are clearly associated with hyperinsulinemia (18). Inflammation was thought to be limited, with only moderate neutrophil infiltration observed in hoof lamellae of horses with laminitis induced using a hyperinsulinemia model (19). However, a study by Holbrook and colleagues showed a marked increase in neutrophil oxidative burst activity in obese hyperinsulinemic horses (20).

The objective of this study was to determine the potential implication of neutrophil activation evidenced by MPO release, following experimental laminitis induction using the prolonged euglycemic hyperinsulinemic clamp (pEHC) model. We hypothesized that as in the BWE model, an increased presence of MPO would be identified in the digital lamellae of horses undergoing pEHC laminitis induction.

## Materials and Methods

### Animals, Laminitis Induction and Sample Collection

Archived formalin fixed–paraffin embedded lamellar tissue samples from 5 horses from a previous experiment were used with approval from the Animal Ethics Committee, The University of Queensland (SVS/506/17). Laminitis was induced using the euglycemic hyperinsulinemic model as described by Asplin and colleagues in three horses (21). Two healthy horses were used as negative controls. An initial intravenous bolus (45 miu/kg) of recombinant human insulin (Humulin-R, Eli-Lilly Australia Pty Lfd) diluted in 50 ml of 0.9% sodium chloride (Baxter Healthcare Pty Ltd) was administered *via* a 14-gauge catheter and was followed by a continuous intravenous infusion of insulin in 0.9% sodium chloride (Baxter Healthcare Pty Ltd) at a fixed rate of 6 miu/kg/min. Additionally, a continuous intravenous infusion of 50% glucose (Baxter Healthcare Pty Ltd) was administered, and the rate was adjusted to maintain euglycemia (4.0 ± 1.0 mmol/L). Blood glucose was measured using a portable glucometer (Accu-Chek Performa, Roche Diagnostics). The Obel scoring system was used to quantify the clinical symptoms (22). After 48 h of pEHC-treatment, horses were subjected to euthanasia, and a minimum of 2 samples of mid-dorsal hoof lamellae were obtained immediately on each horse. Tissues were formalin-fixed for 24 h and transferred to 70% ethanol for 48 h prior to paraffin embedding.

### Immunohistochemical Staining

Histological sections were prepared at 4 μm thickness and mounted on a glass slide following standard technique. Histological sections were deparaffinized and rehydrated following standard protocol: Slides were incubated overnight at 60°C, followed by successive baths in xylene (2 × 5 min), ethanol 100% (2 × 2 min), 95% (1 × 1 min), 70% (1 × 2 min) and phosphate-buffered saline (PBS) (2 × 3 min). Based on preliminary tests, no antigen retrieval protocol and hydrogen peroxide blocking steps were required. Immunostaining of MPO was performed with purified equine MPO and using a rabbit specific horseradish peroxidase/diaminobenzidine ABC detection immunohistochemistry kit (Abcam) (23). Sections were surrounded with a hydrophobic barrier pen prior to addition of protein block solution (PAP Pen, Abcam) for 10 min at 22°C to block nonspecific background staining. After one wash with PBS (1 × 3 min), the primary anti-MPO antibody (rabbit antibody obtained against purified equine MPO) diluted 1:1000 in dilution buffer (20 mM PBS pH 7.4 + 0.5% bovine serum albumin and 0.1% Tween 20) was applied for 1 h at 22°C. Negative control sections were prepared by adding only the dilution buffer without primary antibody. After washing with PBS (3 × 3 min), the anti-rabbit antibody conjugated with biotin (kit) was added to all sections for 15 min at 22°C. Slides were washed again with PBS (3 × 3 min) and the streptavidin-peroxidase solution (kit) was added to all sections for 15 min. After rinsing with PBS (3 × 3 min), the chromogen diaminobenzidine/substrate solution (kit) was added to all the sections and incubated for 5 min. The appearance of MPO labeling (brown colouration) was monitored. The slides were then placed in water (3 × 3 min), followed by addition of hematoxylin-eosin (HE) solution (Merck) for 90 s. The slides were rinsed under tap water for 2 min, and water-soluble mounting media was added and left to dry 24 h in the dark. The sections were assessed using light microscopy with a Zeiss Axioskop microscope, and all photographs were obtained using the same light intensity and shutter speed. Slides of control and treated horses were prepared in parallel and stained simultaneously.

Representative histological sections were selected for each animal and classified in 3 groups: no antibody slides (5); control group (7) and PeHC group (16). Six investigators blinded to the experimental groups semi-quantitatively assessed immunostaining outcomes. For each individual image, MPO labeling was scored by each investigator as follows: no brown staining, weak but localized staining; weak but diffuse staining; medium staining and strong staining. mean scores were calculated for each image (*n* = 28) and calculated for each group.

## Results

### Animals and Clinical Signs

All horses were adult Standardbred geldings retired from racing. The mean ± standard deviation of age and weight was 6 ± 2 years and 492 ± 45 kg, respectively. The pEHC treated horses developed a mild tachycardia, as well as increased digital pulses after 18 to 24 h of pEHC. All pEHC treated horses had clinical signs of Obel grade 1 lameness prior to euthanasia. The control horses did not display any clinical signs of laminitis.

### Immunostaining Intensity

Differences were observed comparing the images coming from the no-antibody slides, the MPO-Stained slides from the healthy horses, and the MPO-Stained slides from the pEHC horses. The mean scores for the MPO labeling were 1,7 (+/– 0,5) for the control horses and 3,4 (+/– 0,5) for the pEHC-treated horses.

### Histopathology and Immunohistochemical Staining

**Figure 1:**
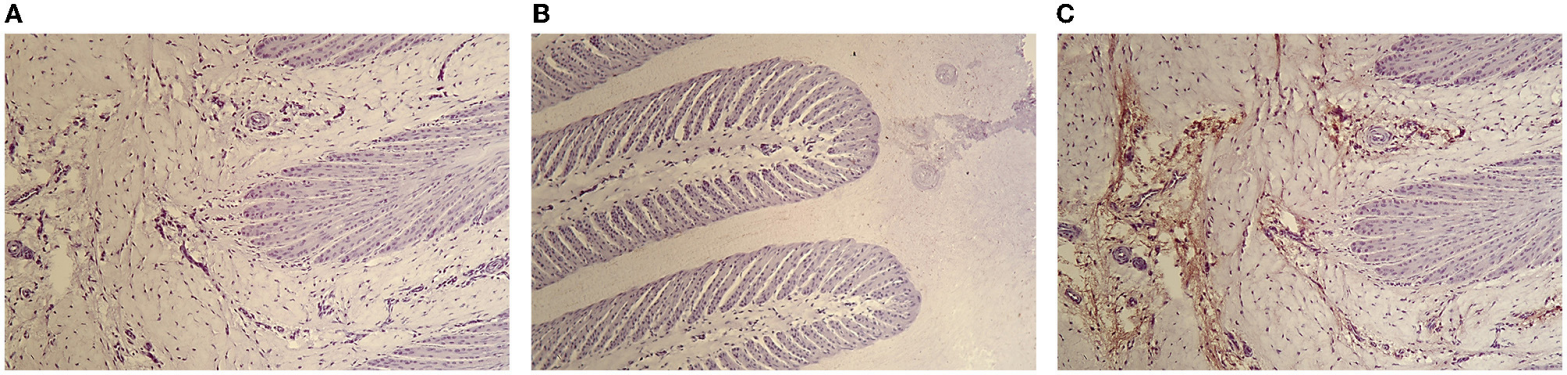
Photomicrograph of the hoof lamellae of a control horse HE staining without primary MPO antibody **(A,B)** and HE staining with complete MPO immunohistochemical protocol **(C)** (× 100).

The Figure 1 shows photomicrographs (× 100) of the lamellae of control horses HE staining without primary MPO antibody (Figures 1A,B) and HE staining with complete MPO immunohistochemical protocol (Figure 1C). Histologic sections of control horses show minimal, localized MPO labeling in the dermal lamellae. The secondary epidermal lamina have rounded tips in control horses. The nuclei of the epithelial basal cells are oval shaped in control horses

**Figure 2:**
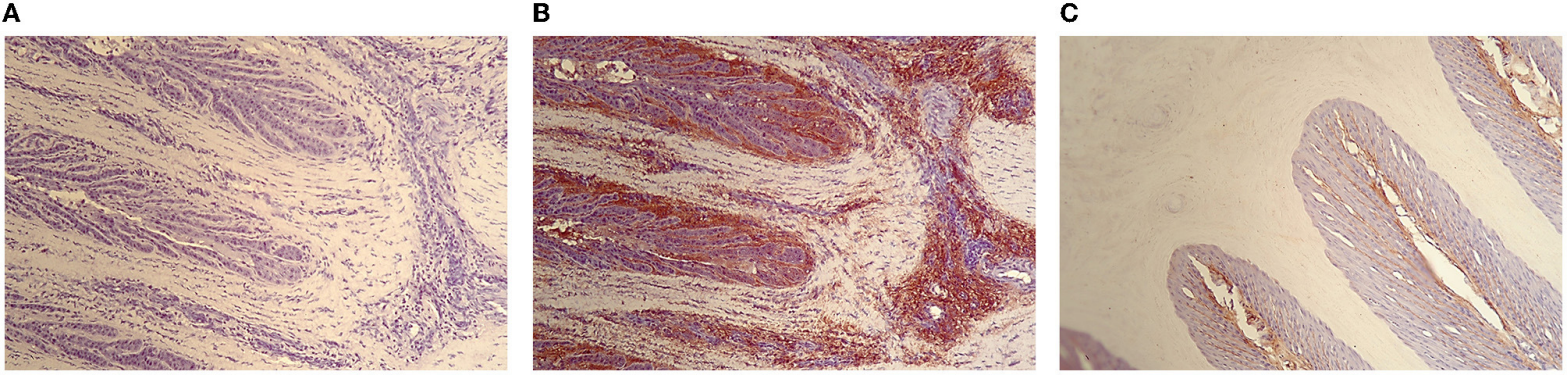
Photomicrograph of the hoof lamellae of a pEHP treated-horse HE staining without primary MPO antibody **(A)** and HE staining with complete MPO immunohistochemical protocol **(B,C)** (× 100).

The Figure 2 shows photomicrographs (× 100) of the lamellae of pEHC-treated horses HE staining without primary MPO antibody (Figure 2A) and HE staining with complete MPO immunohistochemical protocol (Figures 2B,C). Secondary epidermal lamellae have tapered tips, appear elongated and narrow, and are acutely angled on primary epidermal lamellae. An intense diffuse MPO labeling is observed, especially in the interstitial tissue of the dermal lamellae (Figure 2B). A clear demarcation of MPO labeling is observed at the interface of the secondary epidermal and dermal lamellae with absence of MPO labeling on the epidermal side (Figure 2C).

The absence of MPO labeling in negative control slides confirming the absence of non-specific binding of the primary anti-MPO antibody (Figures 1A,B, 2A). In the presence of anti-MPO antibody, MPO labeling is visible in all the samples (Figures 1C, 2B,C).

In the supplemental figure, images show reconstruction of photomicrographs of the dermo-epidermal interface of a control horse and a pEHC-treated stained with HE and anti-MPO. The intensity of the MPO labeling decreases from the dermal side toward the epidermal side of the hoof, so that the MPO labeling is most intense at the base of the primary dermal lamellae and least discernible at their tips.

## Discussion

Laminitis occurring secondary to sepsis is known to result from a significant inflammatory response that includes leukocyte emigration, in particular the neutrophil into the lamellar tissue (1, 2). Horses administered BWE show clinical signs of laminitis and local inflammation with neutrophil activation and MPO release (9, 10). This study highlights the presence of MPO in equine lamellae of horses with insulin-induced laminitis, suggesting that neutrophil activation is also present in this model. All histologic sections of the treated horses presented well-described histological features of the hyperinsulinemia model such as elongated, narrowed secondary epidermal lamellae with tapered tips and acute-angle orientation, as well as rounded and centrally located nuclei (19, 21, 24, 25). Together with the clinical signs of laminitis observed, this confirms that laminitis induction was successful.

Significant positive MPO labeling was observed in all sections of insulin-treated horses. During an excessive neutrophil stimulation, MPO can be released in the extracellular matrix or blood. Therefore, MPO is considered a marker of neutrophil activation or inflammation. Due to its dual activity, MPO can chlorinate, nitrate and oxidize most biological organic molecules, which results in severe tissue damage (26, 27). In addition, MPO can be taken up by endothelial cells, which can be subsequently damaged by its products (28–30). Although the activity of the enzyme is not showed in this study, MPO and the oxidative species derived from its activity could play a role in establishing events leading to laminitis in a variety of ways. The presence of active MPO could perpetuate the lamellar injury.

In previous studies using the pEHC model, a limited number of neutrophils has been observed in lamellar tissue (24). It was concluded that the extent and severity of inflammation in hyperinsulinaemia-induced laminitis are less important than would be expected, when compared to other tissues subjected to similar levels of cellular stress and mechanical compromise (21, 24). However, these conclusions are based on identification of the neutrophil degranulation itself. Our histologic sections agree, with the observation of only rare neutrophils. However, the increased presence of MPO supports the active involvement of neutrophils in the pathophysiology of laminitis. This may be explained by neutrophils degranulation in the bloodstream and MPO diffusion from the circulation to the dermal lamellae. Indeed, the presence of MPO in the bloodstream, skin, and lamellae after laminitis induction using a BWE model was confirmed by Riggs and colleagues (9). Neutrophils, in addition to causing tissue injury when dysregulated, also appropriately respond to damage-associated molecular patterns (DAMPs) signals from other injured tissues. Monocytes/macrophages and endothelial cells can also release a minor quantity of MPO. Additional immunohistochemical staining for calprotectin (using a MAC387 antibody) might be used to support the contribution of neutrophils and monocytes/macrophages in MPO release (17). Another possibility is that neutrophil extracellular traps (NETs) are formed during laminitis, explaining the extensive MPO labeling with almost complete absence of neutrophils. NETs are typically formed to trap micro-organisms, but their formation has also been confirmed during non-infectious disease processes. In human patient, plasma NET parameters such as MPO-DNA complexes were higher in obese patients than in the control group and correlated with body weight, body mass index, waist and hip circumference, glucometabolic parameters, and systolic blood pressure (31). Increased NETosis was also found in type II diabetes patients compared to healthy controls (32).

As in diabetic people where evidence indicates that insulin regulates neutrophil function and that this regulation is in turn related to increased neutrophil chemotaxis and oxidative burst, Holbrook and colleagues showed in horses a marked increase in neutrophil oxidative burst activity in hyperinsulinemic obese horses (20, 33).

However, further experiments are needed to confirm the origin of the MPO presence in lamellar tissue and the possible toxic role that the enzyme could play.

The main limitation is the small number of horses included in the study. This precluded any statistical analysis to complete the histopathological description and qualitative assessment of the immunohistochemical staining. Future studies will focus on the presence of NETs and chlorination residues in lamellar tissues of affected horses as well on the evolution of systemic MPO concentrations.

## Conclusions

This study highlights the presence of MPO in the lamellae of horses with insulin-induced laminitis, supporting a role for neutrophil activation in endocrinopathic forms of laminitis and justifying future research to confirm the link between hyperinsulinemia, neutrophil activation in equine laminitis.

## Data Availability Statement

The raw data supporting the conclusions of this article will be made available by the authors, without undue reservation.

## Ethics Statement

The animal study was reviewed and approved by Approval number: SVS/506/17. The project from which archived samples were used for this experiments was approved by the University of Queensland Animal Ethics Committee (AEC) that monitors compliance with the Animal Welfare Act (2001) and the Code of Practice for the care and use of animals for scientific purposes (current edition). All animals were monitored continuously by the investigators.

## Author Contributions

NS and DS: conceptualization. TF and NS: methodology. CM and AS: investigation. TF and DS: data curation and supervision. NS and GR: writing—original draft preparation. NS, GR, TF, and DS: writing—review and editing. DS: funding acquisition. All authors have read and agreed to the published version of the manuscript.

## Funding

This work was funded by grants from the University of Liege (Lamistem).

## Conflict of Interest

The authors declare that the research was conducted in the absence of any commercial or financial relationships that could be construed as a potential conflict of interest.

## Publisher's Note

All claims expressed in this article are solely those of the authors and do not necessarily represent those of their affiliated organizations, or those of the publisher, the editors and the reviewers. Any product that may be evaluated in this article, or claim that may be made by its manufacturer, is not guaranteed or endorsed by the publisher.

## Supplementary Material

Supplementary Figure S1 Reconstruction of photomicrographs of the dermo-epidermal interface of a control horse stained with HE and anti-MPO (× 100). The dermal side is to the left and the epidermal side is to the right.

Supplementary Figure S2 Reconstruction of photomicrographs of the dermo-epidermal interface of a pEHC-treated horse stained with HE and anti-MPO (× 100). The dermal side is to the left and the epidermal side is to the right.
