# Supplementary figures and images for: Presence of Myeloperoxidase in Lamellar Tissue of Horses Induced by an Euglycemic Hyperinsulinemic Clamp
Source: Front Vet Sci. 2022 Mar 11;9:846835. doi: 10.3389/fvets.2022.846835 (PMC8962398; doi:10.3389/fvets.2022.846835)

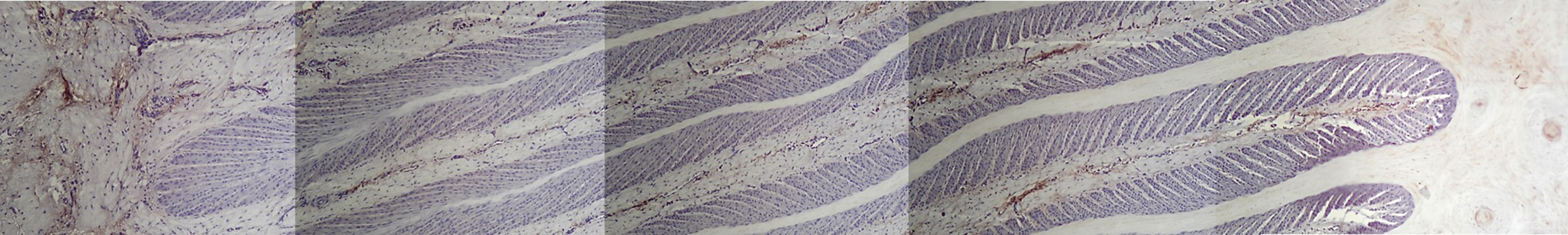

Supplement: Supplementary Figure S1 — Reconstruction of photomicrographs of the dermo-epidermal interface of a control horse stained with HE and anti-MPO (× 100). The dermal side is to the left and the epidermal side is to the right. [file Image_1.JPEG]

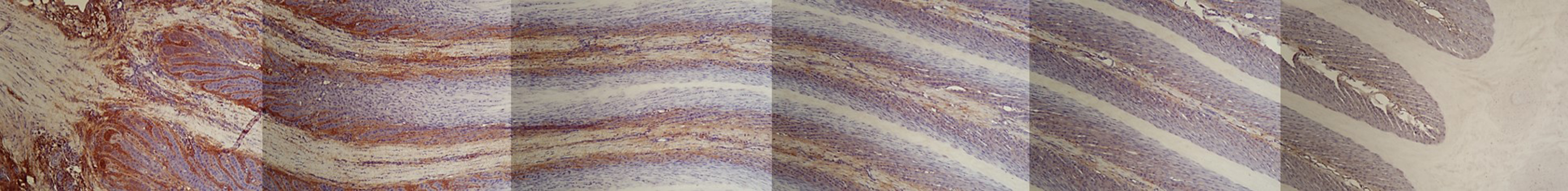

Supplement: Supplementary Figure S2 — Reconstruction of photomicrographs of the dermo-epidermal interface of a pEHC-treated horse stained with HE and anti-MPO (× 100). The dermal side is to the left and the epidermal side is to the right. [file Image_2.JPEG]
